# Dependency Grammar Approach to the Syntactic Complexity in the Discourse of Alzheimer Patients

**DOI:** 10.3390/bs15101334

**Published:** 2025-09-29

**Authors:** Zhangjun Lian, Zeyu Wang

**Affiliations:** College of Humanities and Foreign Languages, Xi’an University of Science and Technology, Xi’an 710054, China; lianzhangjun@xust.edu.cn

**Keywords:** Alzheimer’s disease, dependency grammar, fine-grained grammatical indices, dependency network, syntactic complexity

## Abstract

This study aims to investigate the syntactic complexity in individuals with Alzheimer’s disease (AD) by conducting a comprehensive analysis that incorporates mean dependency distance (MDD), fine-grained grammatical metrics, and dependency network structures. A total of 150 adults with AD and 150 healthy controls (HC) responded in English to interview prompts based on the Cookie Theft picture description task, and the results were compared. The key findings are as follows: (1) The primary syntactic change is a strategic shift from hierarchical, clause-based constructions to linear, phrase-based ones, a direct consequence of working memory deficits designed to minimize cognitive load. (2) This shift is executed via a resource reallocation, where costly, long-distance clausal dependencies are systematically avoided in favor of a compensatory reliance on local dependencies, such as intra-phrasal modification and simple predicate structures. (3) This strategic reallocation leads to a systemic reorganization of the syntactic network, transforming it from a flexible, distributed system into a rigid, centralized one that becomes critically dependent on the over-leveraged structural role of function words to maintain basic connectivity. (4) The overall syntactic profile is the result of a functional balance governed by the principle of cognitive economy, where expressive richness and grammatical depth are sacrificed to preserve core communicative functions. These findings suggest that the syntactic signature of AD is not a random degradation of linguistic competence but a profound and systematic grammatical adaptation, where the entire linguistic system restructures itself to function under the severe constraints of diminished cognitive resources.

## 1. Introduction

Studies on connected speech consistently report significant differences between patients with Alzheimer’s disease (AD) and healthy control (HC) groups across multiple dimensions, particularly within the domain of syntactic complexity, where the specific characteristics remain inadequately defined and a subject of ongoing controversy. On one hand, from a syntactic perspective, several studies (e.g., Appell et al., 1982; Bayles et al., 2000; Kemper et al., 1993; Lyons et al., 1994; Sabat, 1994) suggest that patients with Alzheimer’s disease retain a degree of syntactic competence. Despite significant deficits in the lexical and semantic domains, the fundamental rules governing syntactic structure remain intact, particularly in the construction of simple sentences. Studies (e.g., Bayles et al., 2000; Kemper et al., 1993) have demonstrated that, even with impaired cognitive functions, patients with Alzheimer’s disease are capable of constructing basic subject–verb sentences while maintaining grammatical coherence. However, this preservation is largely confined to simpler structures, as patients with Alzheimer’s disease encounter difficulties with more complex syntactic constructions.

On the other hand, at the lexical level, patients with AD exhibit notable deficits, including discourse deficiencies (Croisile et al., 1996; de Lira et al., 2014; Dijkstra et al., 2004; Nicholas et al., 1985), word-finding and naming difficulties (Appell et al., 1982; Lai & Pai, 2009; H. Y. Liu, 2014), and reduced lexical diversity (Kavé & Dassa, 2018; Orimaye et al., 2017; Williams et al., 2023). With respect to discourse coherence, patients with Alzheimer’s disease face challenges with cohesion and logical continuity, often producing repetitive and fragmented speech patterns (Bose et al., 2021; Dijkstra et al., 2004; Ellis, 1996; Mortensen, 1992).

Furthermore, studies (e.g., Kemper et al., 1993; Tomoeda et al., 1990) suggest that patients with AD tend to avoid nested and subordinate structures, producing simpler sentences that rely on short, coordinated clauses. Patients with Alzheimer’s disease show significant difficulty in generating subclauses, with performance degrading substantially when these clauses are intended to function as components of passive or existential sentences (Kavé & Levy, 2003). Thus, while syntactic rules are preserved in simpler structures, the syntactic generative capacity of patients with Alzheimer’s disease becomes increasingly constrained as task complexity escalates.

It is well established that cognitive decline and semantic memory deficits significantly affect language use in patients with AD (Hier et al., 1985; Kramer & Miller, 2000; Nasiri et al., 2022). As a result, changes in linguistic performance have become key indicators of AD. Investigating the linguistic regression observed in patients with Alzheimer’s disease not only provides valuable insights into medical diagnosis and prevention but also serves as an important method for exploring the intricate relationships between language, cognition, and brain function (J. Liu, 2019).

Early studies primarily evaluated syntactic complexity through its external manifestations, such as sentence length and subjective scales, rather than its internal, fine-grained architecture, such as the specific composition of phrases and the dependency relationships between words. Some studies (e.g., Blanken et al., 1987; Bose et al., 2021) observed a marked reduction in mean sentence length (MSL) in patients with Alzheimer’s disease, while others (e.g., Chapin et al., 2022) found no such correlation using similar indices. Furthermore, Kemper et al. (2001), utilizing rating scales instead of traditional frequency statistics, reported no significant difference between patients with Alzheimer’s disease and healthy elderly individuals in the deterioration of complex syntactic structures. The lack of consensus in evaluations of syntactic complexity has hindered the establishment of a standardized marker distinguishing patients with Alzheimer’s disease from healthy controls. While representing an initial step toward quantifying syntactic complexity, a reliance on such global metrics of production rendered traditional methods insensitive to alterations in the internal, fine-grained architecture of language, thereby failing to capture the specific structural changes that characterize syntactic decline in patients with Alzheimer’s disease. Consequently, investigating syntactic complexity in AD from perspectives beyond sentence length and structural complexity has become increasingly important (Gao & He, 2024).

The limitations of traditional methods that rely on surface-level frequency statistics, such as MSL and counts of specific syntactic structures, have increasingly underscored their inability to accurately characterize the linguistic features of patients with Alzheimer’s disease.

Working memory plays a crucial role in syntactic processing (Alatorre-Cruz et al., 2018; Baddeley et al., 1986; Bayles, 2003; Nasiri et al., 2022), and its decline is considered a significant factor in limiting syntactic competence in patients with Alzheimer’s disease (Kemper et al., 2001; Lee & Kim, 2021; Nasiri et al., 2022). The generation of complex syntactic structures requires substantial cognitive resources, and due to working memory impairments, patients with Alzheimer’s disease encounter difficulties in processing multi-level dependencies and complex syntactic information (Bayles et al., 2000). As a result, individuals with Alzheimer’s disease often rely on simplified syntactic structures, reducing cognitive load by prioritizing basic syntactic rules, which reflects a compromise between maintaining syntactic integrity and simplifying syntactic processing (Kemper et al., 1993; Lyons et al., 1994). Hence, it is hypothesized that working memory capacity may serve as a distinguishing factor in the syntactic complexity between patients with Alzheimer’s disease and healthy controls.

Given the inextricable linkage between working memory, cognitive resources, and syntactic complexity (Nasiri et al., 2022), a more exhaustive interrogation of the nuanced linguistic manifestations in Alzheimer’s disease constitutes a scientific imperative (Gao & He, 2023). In a departure from orthodox methodologies predicated primarily on sentential length and gross structural analyses, the dependency grammar paradigm conceptualized by Tesnière (1959) posits a more theoretically robust and dynamic framework for the present inquiry. Emanating from this paradigm, Mean Dependency Distance (MDD) serves to quantify syntactic complexity via the measurement of the mean linear distance separating syntactically co-dependent lexical items. Augmented distances are understood to exact a greater toll on working memory—a consequence of the cognitive necessity to maintain antecedent words in active storage pending the appearance of their syntactic dependents—thereby elevating the cognitive expenditure requisite for successful sentence resolution. Consequently, MDD emerges as a direct proxy for the cognitive exertion entailed by the parsing of syntactic structures.

Secondly, to complement the macro-level assessment afforded by MDD and to ascertain the specific manifestations of syntactic alteration, a second tier of analysis concentrates on micro-level syntactic features. Through the computation of a suite of fine-grained syntactic indices via the TAASSC software (Kyle, 2016), a precise quantification and delineation of the subtle distinctions between grammatical units is achieved. This procedure is not merely designed to capture the concrete complexities of linguistic structure, but more critically, to elucidate the profound nexus between cognitive load and specific syntactic capacities, such as the processing of multi-tiered dependencies, thereby substantiating macro-level findings with robust micro-level evidence.

Building upon the two preceding quantitative analyses, the third tier of analysis pivots to a topological examination of syntactic structural relations, an approach intended to transcend isolated, linear metrics and to probe the entire architectural system of syntax constituted by interconnected lexical units. To this end, the methodology employs the Pajek (de Nooy et al., 2018) software to calculate and introduce a suite of key network topological indices for the in-depth quantification of the structural function of each lexical node: Betweenness Centrality (C_B_), which elucidates a node’s function as a critical syntactic conduit for maintaining global connectivity; Closeness Centrality (C_C_), which quantifies its integrative capacity as a network hub for efficiently reaching all other constituents; and the Clustering Coefficient (C), which delineates the degree to which a node anchors a highly cohesive, localized syntactic domain amenable to modular processing. By focusing on the network of dependencies rather than on isolated structural analyses, this systemic perspective therefore affords a more dynamic and methodologically robust approach to capturing the challenges faced by Alzheimer’s patients in processing complex syntax (H. Liu, 2008b).

Although prior investigations into AD have discerned subtle variations in syntactic complexity, such findings have frequently been fragmentary, lacking a comprehensive and integrated theoretical perspective (Emery, 2000). Therefore, predicated on the theoretical framework of dependency grammar, the present study aims to interrogate the alterations in syntactic complexity among patients with Alzheimer’s disease through an integrated analysis of fine-grained grammatical indices, mean dependency distance, and dependency networks. A dependency treebank has been constructed from transcriptions of oral recordings from both the AD and HC cohorts. This study seeks to address the following two research questions to provide preliminary insights into the aforementioned limitations:

Q1: Can MDD, fine-grained syntactic indices, and dependency networks account for changes in the syntactic complexity of AD?

Q2: If such changes are identified, what are the specific linguistic differences between the AD and HC groups?

## 2. Materials and Methods

### 2.1. Corpus

The corpus materials utilized in this study originate from the DementiaBank clinical corpus, which was developed through research on Alzheimer’s disease and related dementias at the University of Pittsburgh School of Medicine. The original data was subsequently processed and converted into a syntactically annotated treebank. This DementiaBank clinical corpus comprises spoken transcripts from individuals diagnosed with Alzheimer’s disease, mild cognitive impairment, and various other dementias, as well as from cognitively healthy controls. Participants responded in English to interview prompts based on the “Cookie Theft” picture description task (Goodglass & Kaplan, 1983), a standardized tool widely used for assessing language deficits in individuals with AD and aphasia (Rohrer et al., 2010). The participants in the Alzheimer’s Disease (AD) group (44 males, 106 females) had an age range of approximately 56–85 years, while the Healthy Control (HC) group (39 males, 111 females) comprised individuals aged 61 to 82 years, with no reported neurological or cognitive impairments. The Mini-Mental State Examination (MMSE) scores for the AD group ranged from 3 to 20, and all were diagnosed with “Probable AD.” The MMSE scores for the HC group ranged from 27–30. Given that the DementiaBank corpus is longitudinal and contains multiple recordings for each participant over several years, the selection of a single recording was a deliberate methodological choice. To enhance the representativeness of our sample and the generalizability of our findings, we prioritized maximizing the number of unique individuals rather than using multiple data points from a smaller set of participants, which would reduce sample independence. Therefore, we consistently selected the first available “Cookie Theft” task recording for each participant. It was also confirmed that the average recording lengths were comparable between the AD and HC groups, ensuring that the volume of transcribed text did not introduce a potential confound to the analysis.

The study focuses on the syntactic features of connected speech in patients with Alzheimer’s disease, and irrelevant transcription symbols and interjections were excluded during the data cleaning process. Additionally, the transcriptions were segmented into independent speech units based on dialogue boundaries and intonation contours. Incomplete utterance (e.g., “the woman is washing …”), sentence fragments (e.g., “water spilling out of the sink”), and complete sentences were treated as independent utterances. Additionally, independent noun phrases (e.g., “and the window”), non-restrictive verb phrases (e.g., “snitching cookies”), and prepositional phrases (e.g., “of the sink”) were also considered independent discourse units and included in the analysis process (Hier et al., 1985).

### 2.2. Data Collection

The concept of dependency distance (DD) refers to the syntactic separation between a dependent and its governor in a sentence, and it has been widely adopted as a metric to evaluate the structural complexity of sentences (Jiang & Ouyang, 2018; R. Hudson, 2010; H. Liu, 2008a; Futrell et al., 2015). Shorter dependency distances often indicate simpler sentence structures, while longer distances suggest more intricate syntactic arrangements. In this study, the MDD for each text was extracted using the method proposed by H. Liu (2009). Specifically, the MDD of a sentence can be calculated as:

(1) $$MDD\left(sentence\right)=\frac{1}{n-1}\sum_{i=1}^{n}DDi$$

**Figure 1:**
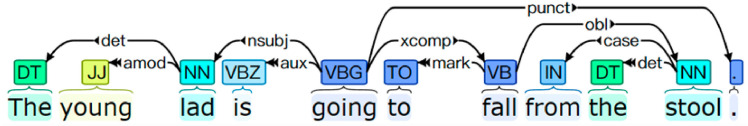
Dependency analysis of the sample sentence.

In this formula, n represents the total number of words in the sentence, while DDi denotes the dependency distance of the *i*-th syntactic connection. Using this formula, the dependency structure and MDD of the sample sentence “The young lad is going to fall from the stool.” presented in Table 1 and Figure 1, can be derived as follows:

$$MDD\left(sample\;sentence\right)=\frac{2+1+2+1+1+2+2+1+3+6}{10-1}=2.33$$

To calculate the MDD of the entire text, the second formula uses n to represent the total word count and s for the total number of sentences. It should be emphasized that the calculation excludes the dependency distances associated with punctuation marks and root tags.

(2) $$MDD\left(text\right)=\frac{1}{n-s}\sum_{i=1}^{n}DDi$$

During the corpus cleaning process, a Python (3.8.0) script, utilizing the Stanza (1.8.2) natural language processing package, was employed to remove interjections (such as “mhh”, “umm”, “um”) and symbols like “<”, “>”, “&”, and “+”. To first confirm the parser’s reliability on this specific dataset, its performance was validated against a 100-sentence manually annotated gold standard, achieving both Unlabeled Attachment Score (UAS) and Labeled Attachment Score (LAS) above 85%. To ensure reliability, a 10% subset from both the AD and HC groups was independently annotated by two linguistics researchers. Inter-annotator agreement was subsequently assessed using Cohen’s Kappa, a metric that corrects for chance agreement. Recognizing the distinct linguistic characteristics of each group, calculations were performed separately. This analysis yielded a κ value of 0.84 for the AD group and a κ value of 0.76 for the HC group, indicating “almost perfect” and “substantial” agreement, respectively (Landis & Koch, 1977). All identified disagreements were adjudicated via a systematic protocol (Oortwijn et al., 2021), wherein a third linguistics researcher guided a diagnostic process to ascertain the root cause of each conflict. Crucially, the resolutions derived from this process informed the iterative refinement of the annotation guidelines, ensuring the consistent application of a robust annotation scheme throughout the corpus. Pajek (1.0.0), a widely used software for network analysis and visualization, was then employed to construct and analyze dependency treebanks. Following the cleaning process, the two resulting corpora were used to facilitate network analysis with CreatePajek, a tool within Pajek for network visualization and structure analysis. For syntactic feature analysis, TAASSC (1.3.8), a computational linguistics-based tool for automatic analysis of sophistication and complexity, was used to process the input corpus and provide syntactic descriptions of variations in MDD. This tool automates the extraction of fine-grained indices related to specific syntactic structures, facilitating the selection of indices aligned with the research objectives. Subsequently, SPSS (IBM Corp., 2020) software (Version 27.0) was used to perform the statistical analyses, which included calculating the MDD and other relevant indices for both groups and conducting various statistical validation procedures.

### 2.3. Statistical Analysis

The Shapiro–Wilk test indicated that the MDD data from both the AD group (*p* = 0.274, *p* > 0.05) and the HC group (*p* = 0.075, *p* > 0.05) followed a normal distribution. An independent t-test was subsequently conducted to assess the differences between the AD and HC groups. To explore the relationship between MDD and syntactic features, Pearson correlation analysis was performed, excluding any correlations that were either not significant (*p* ≥ 0.05) or yielded minimal values (r < 0.100). The remaining indices underwent multicollinearity checks, with those exhibiting high variance inflation factors (VIF ≥ 5) being excluded. The syntactic features most strongly correlated with MDD (r ≥ 0.100) were retained for further analysis (Kyle & Crossley, 2018). Stepwise regression analysis was then applied to identify the syntactic indices most strongly associated with MDD, as shown in Table 2. The selected indices typically represent syntactic characteristics that exhibit the most prominent relationships with MDD.

### 2.4. Syntactic Analysis

A stepwise regression analysis was subsequently employed to identify the syntactic indices most significantly correlated with MDD. The selected indices typically reflect syntactic features that demonstrate the strongest associations with MDD. Following the conversion of the AD and HC treebanks into dependency networks, an analysis was conducted on the word vertices, encompassing both parent and dependent nodes, along with their corresponding structures. This section provides a comparative analysis of the key vertices and dependency structures within the AD treebank and the reference HC treebank.

## 3. Results

### 3.1. MDD Variation Across the AD and HC Groups

To begin with, the distributions of MDD in the AD and HC groups were compared to examine differences in syntactic complexity. The AD group exhibited lower MDD, whereas the HC group demonstrated higher MDD. This macro-level analysis revealed a significant difference in MDD between the two groups, indicating disparities in syntactic complexity. These differences were further examined through an independent t-test with the following findings.

As presented in Table 3, the MDD of the AD group was 2.56 (rounded to two decimal places), lower than the HC group’s MDD of 2.67. Table 3 further reveals a significant difference in MDD between the two groups. The Levene’s test yielded an F-value of 0.256 and a *p*-value of 0.614 (*p* > 0.05), confirming the assumption of equal variances, with no significant difference in variance between the two groups. Under the assumption of equal variances, the two-tailed *t*-test yielded a *p*-value of 0.001 (*p* < 0.05), indicating that the mean MDD of the AD group was significantly lower than that of the HC group.

### 3.2. Grammatical Indices of the AD Group in Reference to Those of the HC One

Before conducting the stepwise regression, indices that did not demonstrate a significant correlation with MDD (*p* ≥ 0.05 or r < 0.100), as well as those exhibiting multicollinearity (VIF ≥ 5), were excluded. The remaining indices were subsequently included in the regression model. The model identified 15 significant indices of MDD in the AD group, nine of which were associated with noun phrase extension, as outlined in Table 4. The model accounted for 69.9% of the variance in MDD (R^2^ = 0.699, adjusted R^2^ = 0.665).

The predictive model for MDD in the HC group includes eight indices, as presented in Table 5. This model accounts for 66.2% of the variance in MDD for the HC group (R^2^ = 0.662, adjusted R^2^ = 0.643).

### 3.3. Dependency Network of the AD Treebank in Reference to Those of the HC One

**Figure 2:**
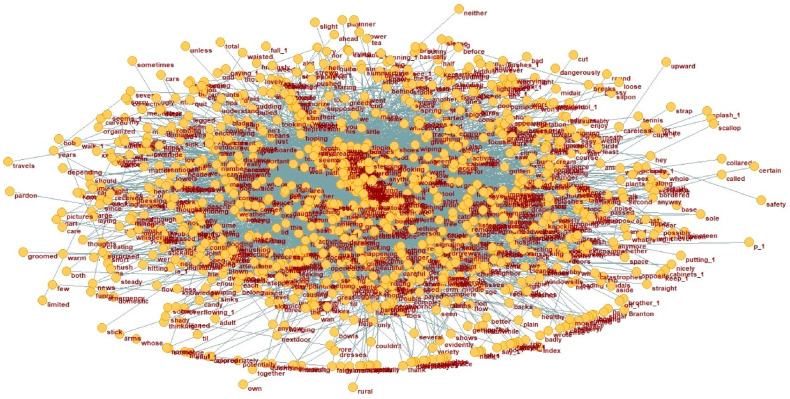
Dependency network of AD group (N1).

**Figure 3:**
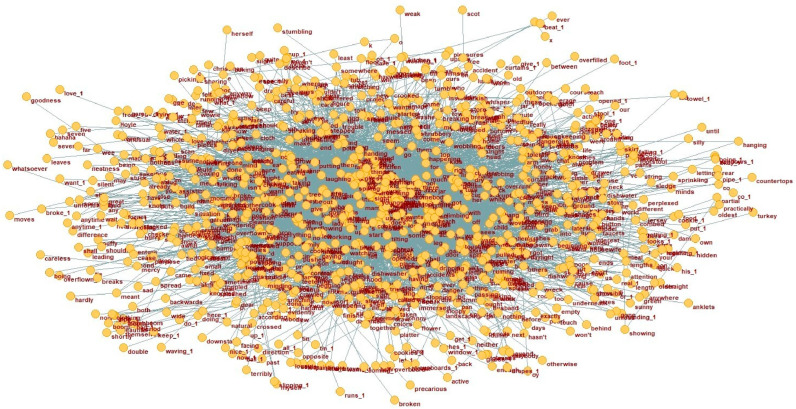
Dependency network of HC group (N2).

A language network consists of vertices representing words and edges illustrating the binary relationships between them. In dependency grammar, an edge signifies the asymmetric relation between a head word and its dependent, with the direction of dependency being crucial (R. A. Hudson, 2007; R. Hudson, 2010; H. Liu, 2009). Such a network provides an effective framework for modeling linguistic structure and meaning through both graphical and quantitative analyses. At the macro level, the distribution of dependency distances was analyzed across the AD and HC corpora. This section offers a comparative analysis of key vertices and dependency structures in the AD treebank and the reference HC treebank. After converting the AD and HC treebanks into dependency networks, we examined the word vertices—both parent and dependent nodes—and their associated structures. This analysis sheds light on the underlying framework of dependency distances and the functional distinctions between the two groups. Using Pajek, the treebanks were transformed into network representations, which were then analyzed to highlight the dependency relationships in the AD network that distinguish it from the HC network. The dependency networks for the AD and HC groups are shown in Figure 2 and Figure 3, respectively.

A subset of the results, particularly the top 50 dependency pairings, is presented in Table 6. The data show that the most central dependency pair in the AD network is “there-x,” with “there” serving as the parent node, exhibiting a higher degree of centrality compared to the HC network. In contrast, the predominant dependency pair in the HC network is “it-x,” where “it” serves as the parent node. The dependency relations within the HC network are comparatively more evenly distributed, reflecting a broader structural scope.

**Figure 4:**
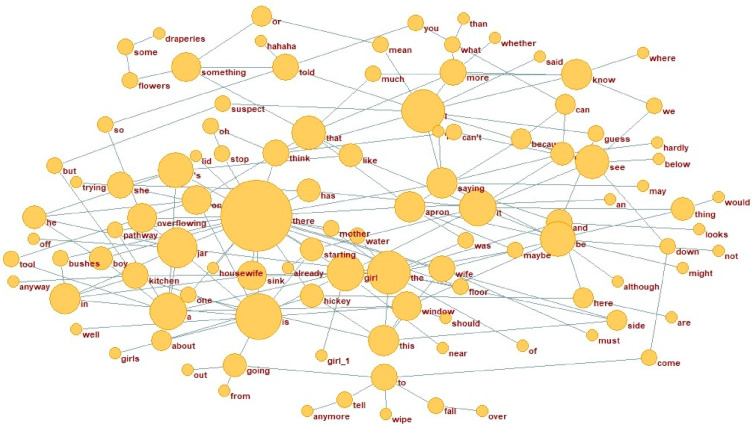
Top 200 cross difference of AD network (N3).

**Figure 5:**
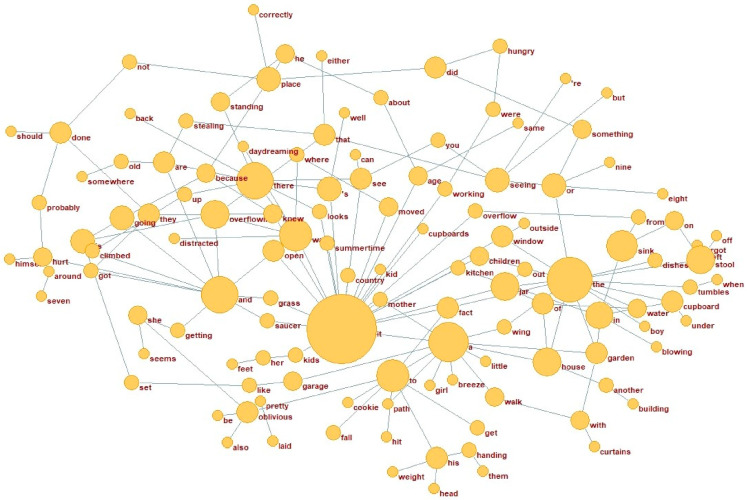
Top 200 cross difference of HC network (N4).

The preceding analysis provides a description of the top 50 central dependency pairs within the AD and HC networks, emphasizing their structural significance. To further investigate this aspect, the top 200 dependencies in the AD network were selected for comparison with the HC network, which served as the reference. This comparison specifically aimed to examine the structural differences between the two networks. The networks and weighted vertices are presented in Figure 4 and Figure 5.

The weighted degree of vertices depends on both vertex degree and edge values, together reflecting their centrality within the network. In the AD network, the words with the highest vertex degrees are “there,” “is,” and “I” which perform key syntactic functions in sentence structure. In contrast, the word “it” ranks highest among the top 30 weighted vertices in the HC network, with its adjacent vertices forming a structure primarily centered on statements and judgments, as illustrated in Table 7.

## 4. Discussion

The regression model indicates that syntactic patterns in individuals with Alzheimer’s disease do not reflect a random loss of linguistic competence. Rather, they reveal a systematic reorganization that emerges under limited cognitive resources to maintain communicative efficiency. This reorganization involves a shift from clausal, hierarchical complexity to phrasal, linear complexity. The shift is driven by the need to minimize reliance on working memory, as quantitatively reflected in variations in MDD. Central to this thesis is the conceptualization of the observed syntactic simplification in patients with Alzheimer’s disease, defined by a marked linguistic reduction in complex sentences with nested structures and explicit conjunctions, as an essential cognitive avoidance strategy. Such constructions impose a substantial cognitive load, necessitating the maintenance of long-distance syntactic dependencies within working memory. In contrast, Noun Phrases, especially elaborated variants, though capable of conveying substantial semantic information, are inherently more linear and localized in their syntactic architecture. Information is concatenated around the nominal head via pre- and post-modifying elements (e.g., adjectives, prepositional phrases), a configuration that obviates the need to construct complex dependency arcs spanning multiple clauses. Consequently, for individuals with AD, whose working memory resources are severely compromised, prioritizing the deployment of elaborated noun phrases over the generation of complex clausal structures represents a more cognitively economical communicative strategy (Almor et al., 1999).

The MDD data herein lend robust empirical support to the foregoing assertion. As shown in Table 4, the frequency of prepositional phrases functioning as post-modifiers within noun phrases demonstrates a significant positive correlation with MDD (B = 1.505). This finding reveals a seeming paradox: notwithstanding a global pursuit of syntactic simplification, the local strategy employed by patients—namely, the elaboration of nouns with prepositional phrases—extends the linear distance between critical constituents (e.g., subject and predicate), consequently elevating the MDD (Gibson, 1998). A perspicuous illustration of this mechanism is found in Example (1a), which is not a complete clause but rather a single, massively elaborated noun phrase built from the nominal head “sight” followed by a linear concatenation of five prepositional phrases. This structure perfectly instantiates the strategy of phrasal elaboration. Instead of generating a hierarchically complex sentence with multiple clauses, the patients with Alzheimer’s disease anchor all information to a single noun. This linear, additive strategy minimizes the need for complex syntactic planning that would tax working memory. The high MDD value is a direct consequence: a long chain of modifiers intervenes between the subject head and any potential predicate, stretching the dependency distance. Similarly, (1b) builds its subject noun phrase and its predicate complement using participial phrases. These function as reduced relative clauses, a syntactic choice that is less demanding than producing a full relative clause with an explicit relative pronoun (e.g., which is spilling). In both cases, patients with AD accept the cost of increased linear distance, as quantified by the high MDD, to circumvent the greater cognitive burden of hierarchical embedding. This balance, wherein linear distance is exchanged for hierarchical simplification, constitutes the very crux of the syntactic restructuring strategy observed in AD. Patients preferentially incur the cost of an augmented dependency distance between a subject and its predicate rather than generate a hierarchically more complex relative or adverbial clause to convey equivalent information. This indicates that, for patients with AD, the processing of deeply embedded hierarchical structures is more resource-intensive for working memory than the processing of long-distance linear dependencies.

(1) a. The sight through the window of green grass, bushes, window from the house with drapes. (AD Treebank, MDD = 2.71)

b. The pouring water spilling from the sink is just a careless mistake caused by the woman. (AD Treebank, MDD = 2.56)

Further substantiating this account, and in contrast to the preceding finding, the number of verbal modifiers per noun is negatively correlated with MDD (B = −1.018). This outcome reinforces the centrality of the localization principle. It suggests that when modifying elements are verb-affiliated, they exhibit a tendency to cluster in close proximity to the verbal head, thus contracting the relevant dependency distances. The syntactic production mechanism in AD, whether engaged in the elaboration of nominal phrases or the organization of verbal phrases, appears to operate under a unitary principle: the local satisfaction of syntactic dependencies is maximized to circumvent the need for long-distance retrieval operations that tax working memory.

The syntactic profile of patients with Alzheimer’s disease revealed herein transcends a simplistic characterization as mere “simplification” or “error.” It constitutes a profound grammatical and adaptive reorganization, whereby the patient’s linguistic system transitions from a mode that preferentially employs clauses to construct complex, hierarchical information to one that relies on phrasal elaboration for the linear organization of information. This shift directly projects working memory constraints onto language production, with MDD serving as a precise index of cognitive load and revealing the logic underlying these syntactic strategies.

The regression model presented in Table 4 further elucidates the cognitive balance mechanisms driving syntactic restructuring in AD, namely, a systematic arbitration between long-distance, hierarchical dependencies and short-distance, local dependencies. The quantity of subordinating conjunctions per clause exhibits a significant positive correlation with MDD (B = 0.879), indicating that these conjunctions are the syntactic markers for the construction of complex, hierarchical syntactic structures. Their function is to embed one clause within another, thereby invariably generating long-distance dependency arcs that span multiple syntactic constituents. Example (2a), … fall [because] the stool is …, offers a clear illustration of this principle. The introduction of the conjunction “because” establishes a long-distance dependency between the main clause predicate “fall” and the subsequent adverbial clause of reason. This necessitates that the speaker simultaneously activates and maintains two propositional events and their syntactic linkage within working memory, resulting in an elevated cognitive load that is directly manifested as a higher MDD value (MDD = 3.0). The positive coefficient (B = 0.879) can therefore be interpreted as a quantification of the cognitive cost appended to the syntactic operation of clause-building. For patients with Alzheimer’s disease, whose working memory resources are circumscribed, the systematic avoidance of such high-cost operations is the foundational cause for the “simplified” character of their linguistic output.

(2) a. And he’s going to fall [because]_subordinating conjunction_ the stool is, it’s tilted too much. (AD Treebank, MDD = 3)

b. She’s got [short]_adjective_ skirt and a jersey sweater. (AD Treebank, MDD = 2.56)

c. The mother was drying [her dishes]_direct object_. (AD Treebank, MDD = 1.67)

In contradistinction, the model reveals two significant negative correlations: the number of adjectival modifiers per noun (B = −0.790) and the number of direct objects per clause (B = −0.299) are both inversely proportional to MDD. These two findings converge to indicate that the compensatory strategy in the linguistic reorganization of patients with Alzheimer’s disease is the prioritization of local dependencies. An adjective, as a pre-nominal modifier, enters into a syntactic relationship with its head noun that is, in essence, highly localized. As demonstrated in example (2b), … [short] skirt …, the modifier “short” is immediately adjacent to its head noun “skirt,” entailing a minimal dependency distance. Such intra-phrasal modification exacts a negligible toll on working memory. The deeper implication of the negative coefficient (B = −0.790), therefore, is that it signifies more than simply reduced adjective use; rather, it reveals that this local dependency pattern is critical for maintaining a low MDD and, by extension, a low cognitive load. When a sentence relies more heavily on this mode of local modification for semantic enrichment, its overall mean dependency distance is correspondingly reduced. Similarly, the direct object, as a complement to the verb, typically follows it immediately, forming a stable and highly local verb-object constituent. The dependency in example (2c), … drying [her dishes] …, is nearly minimal (MDD = 1.67). This structure represents the most fundamental and economical predicate-core construction in language. The negative coefficient (B = −0.299) indicates that the linguistic output of patients with Alzheimer’s disease gravitates toward a reliance on such simple predicate structures, wherein the verb directly governs its object, rather than on more complex configurations that would necessitate the introduction of multiple clauses or indirect arguments. Overall, the grammar of AD is not fundamentally impaired; instead, it avoids long-distance, cross-clausal dependencies, such as those introduced by subordinators, while favoring short-distance dependencies that reduce MDD.

The regression model in Table 4 offers critical insight into syntactic attrition in AD. A pivotal and seemingly paradoxical finding is that the model predicting MDD for the AD cohort incorporates a greater variety of predictive variables than its counterpart for the HC group. This proliferation of predictors must not be misconstrued as indicative of a more complex or robust system. On the contrary, it is the statistical signature of systemic fragmentation and the erosion of a centralized, efficient grammatical core.

In a healthy linguistic system, syntactic complexity, indexed by MDD, is governed by a small set of potent and interconnected syntactic operations, primarily related to the construction of complex, hierarchical clauses; subordinate and embedded structures are the principal determinants of syntactic complexity for HCs. By contrast, the linguistic system in AD, constrained by reduced working memory, lacks such centralized control. Consequently, their sentence planning devolves into a more piecemeal and localized process (Mortensen, 1992). Syntactic complexity is no longer contingent upon a few high-level syntactic choices but is rather the aggregate outcome of numerous inefficient and mutually disjointed local compensatory strategies. These strategies, which may include the deployment of specific prepositional phrases or a greater reliance on simple coordinators, each contribute minimally and independently to variance in syntactic complexity, thus necessitating distinct predictors within the statistical model. The complexity of the AD model, therefore, reflects not syntactic richness, but the decomposition of a unitary system into a series of dissociated, lower-level production processes.

This interpretation is further corroborated by the goodness of fit for the AD model (R^2^ = 69.9) relative to the HC model (R^2^ = 66.2). The higher explanatory power suggests that the linguistic output of patients with Alzheimer’s disease is more rigidly determined by its intrinsic constraints. Healthy controls, possessing greater cognitive resources, exhibit more linguistic optionality; their syntactic choices are influenced not only by cognitive resources but also by a variety of pragmatic, stylistic, and discursive factors that a purely structural model cannot fully capture. The linguistic choices of patients with AD, however, are guided in a near-deterministic fashion by the constraints of their compromised cognitive resources. Their reliance on piecemeal strategies reflects not creative alternatives but obligatory responses to cognitive impairment. That a regression model based on syntactic features achieves greater explanatory power for this population is, in itself, potent evidence of their syntactic system being locked in by cognitive deficits. The cognitive decline inherent to AD thus predicts, in a quantifiable manner, the very syntactic paradigm through which patients organize and express information.

In contrast to the cognitively constrained syntactic system of patients with Alzheimer’s disease, the regression model for HC reveals a more flexible and dynamic grammatical system governed not by the principle of cognitive economy (Rosch, 1978), but by communicative intent (Sperber & Wilson, 1986). A core feature of this system is the ability of HC group to freely deploy cognitive resources to construct high-cost yet high-yield complex syntactic structures, thereby achieving more precise and nuanced semantic expression.

As shown in Table 5, the MDD in the HC group is positively correlated with the number of dependents per clause (B = 0.401). This quantitative finding is a direct reflection of the productivity of a healthy linguistic system. The linguistic output of HCs contains a substantial number of syntactically more complex hierarchical structures, such as the object clause …[that’s very important and sometimes typical] in example (3a) and the relative clause …[which is overflowing onto the floor] in (3b). These clauses, functioning as dependent components of higher-level structures, necessarily introduce long-distance dependencies and thus elevate the overall MDD. This demonstrates that for cognitively unimpaired individuals, increasing MDD in exchange for greater informational density and logical precision is a normative syntactic strategy in service of communicative goals.

(3) a. I think [that’s very important and sometimes typical]_object clause as a clausal dependent_. (HC Treebank, MDD = 2.78);

b. There is water coming out of a faucet into a basin [which is overflowing onto the floor]_relative clause as a clausal dependent_. (HC Treebank, MDD = 3);

c. The boy is getting the cookies out of the cookie jar [and]_conjunction_ getting ready to fall off the stool which he’s standing on. (HC Treebank, MDD = 3.29);

d. There’s a cup [and]_clausal coordinating conjunction_ she dried two cups and a dish. (HC Treebank, MDD = 2.67).

Of greater theoretical interest is the model’s nuanced differentiation of the effects of various conjunction types, which discloses the syntactic flexibility inherent to the HC cohort. First, the total number of conjunctions per clause is positively correlated with MDD (B = 0.842). This is principally because conjunctions are frequently employed to link coordinate predicates or clauses, thereby expanding the core architecture of the sentence. As illustrated in example (3c), the conjunction “and” links two complex verb phrases, getting the cookies… [and] getting ready to fall…. This operation significantly increases the sentence’s length and structural complexity, culminating in a higher MDD (MDD = 3.29). When the analysis is focused specifically on coordinating conjunctions, however, their frequency exhibits a negative correlation with MDD (B = −0.452). This seemingly contradictory result precisely reflects the dual functionality of the HC grammatical system. When coordinating conjunctions are utilized to link low-level noun phrases (e.g., two cups and a dish in example (3d)) rather than entire clauses or predicates, they establish highly localized dependencies. Such intra-phrasal coordination, similar to adjectival modification, is a cognitively low-cost operation that increases the number of minimal dependency arcs, thereby lowering overall MDD. This facility for fluidly alternating between distinct syntactic strategies is a direct manifestation of a healthy cognitive system at the level of language production.

A cross-group comparison of the regression models reveals a fundamental, asymmetrical difference between the AD and HC cohorts at the level of the syntactic system. The crux of this difference lies in the altered relationship between cognitive resources and the efficiency of syntactic output. Specifically, the HC grammatical system exhibits a “high-gain” characteristic, whereas the AD system degenerates into an attenuated system. The empirical basis for this assertion derives from the markedly divergent regression coefficients for three clause-building metrics across the two models: open clausal complements, subordinating conjunctions, and total conjunctions. Within the HC group, these three metrics all display exceptionally high positive coefficients (B = 0.948, B = 0.936, and B = 0.842, respectively), indicating that their grammatical system efficiently converts these syntactic elements into global syntactic complexity. Each deployment of a clause functions as a high-return investment of cognitive resources, significantly elevating MDD in exchange for more finely grained semantic and logical hierarchies (Kemper et al., 1993). In the model for the AD group, however, while the direction of the association for these metrics remains consistent, their regression coefficients are significantly attenuated (B = 0.433, B = 0.879, and B = 0.510, respectively). This attenuation in the coefficients signifies more than a simple reduction in frequency of use; it reveals a deeper, systemic impairment in the clause generation capacity of patients with AD. Even on the occasions they produce such structures, their contribution to overall sentence complexity is markedly diminished, suggesting that such operations can only be executed within contexts that are already structurally simplified. This is not a strategic simplification but rather an intrinsic attrition of syntactic generative capacity (Chapin et al., 2022).

An analysis of lexical diversity, as measured by the Type-Token Ratio (TTR), provides further corroboration for the aforementioned systemic disequilibrium in the discourse planning capacities of patients with AD. The TTR for the AD group (0.1007) was slightly higher than that for the HC group (0.0868). This anomalous statistical result is, in fact, a statistical artifact of severe discourse fragmentation. As illustrated by the empty phrase in example (4a), [There is, that this], and the complete perseveration in (4b), [you got to get the, you got to get the], the linguistic output of patients with AD is replete with hesitations and non-productive self-corrections (Dijkstra et al., 2004; Nicholas et al., 1985). Owing to their minimal length, these fragmented utterances cause function words and other unique types to carry a disproportionately high weight relative to the low total number of tokens, thereby computationally inflating the TTR value. This higher TTR is thus an inverse indicator of their inability to construct fluent, coherent sentences.

(4) a. [There is, that this]_empty phrase_ should be a window. (AD Treebank)

b. No you’ve got to, [you got to get the, you got to get the]_complete repetition_ the thing out of the out of the… (AD Treebank)

This deficit in discourse planning directly results in a retreat to a syntactic safe mode, manifesting as an over-reliance on the existential there-construction. According to Systemic Functional Grammar, the existential process, which merely describes the concept of existence without involving complex actions or mental states, is the most cognitively economical mode of predication (Halliday, 1994). The existential there-construction provides a low-cost syntactic framework for patients with Alzheimer’s disease. As a semantically light function word, there assumes the role of syntactic subject, relieving the individuals with Alzheimer’s disease of the cognitive load of conceptualizing and generating a complex nominal subject. Instead, the core entity to be expressed is simply inserted into this prefabricated syntactic slot (Breivik, 1981; Wang, 2007). This drastically reduces the cognitive load of syntactic planning and is emblematic of their general trend toward linguistic simplification. In contrast, while healthy controls also use function words, their preferred reduplicative it-construction is, in cognitive terms, the functional inverse of the existential there. The “it” used by the HC group serves not to simplify, but to manage complexity. Here, “it” functions as a syntactic placeholder that anticipates and points to a postposed, more complex clausal argument. The use of this structure presupposes the speaker’s ability to pre-plan the entire complex proposition and is a sophisticated syntactic operation in service of advanced informational and pragmatic goals. It reflects a healthy linguistic system with ample cognitive resources, capable of flexibly deploying syntactic options to achieve communicative ends.

Subsequent to the revelation by MDD of a contraction in the linear dimension of syntactic production in AD, an analysis of the topological structure of the dependency network provides corroboration from the novel dimension of node importance, further elucidating the simplified morphology the AD linguistic system adopts to accommodate cognitive decline. An analysis of the 300 most important dependency relations in the local network reveals that the AD group’s network exhibits a more concentrated profile of dependency types. This represents a drastic contraction of syntactic diversity, whereby a few functional dependency relations appear with extremely high frequency, while more complex dependency types become sparse. This network centralization is a direct macro-statistical projection of the syntactic-level phenomena. Specifically, it reveals that the linguistic output of patients with Alzheimer’s disease has severely regressed to and become fixated on existential structures at the syntactic-semantic level (Zhou & Wang, 2012, p. 65). Existential sentences and copular structures are foundational, low-cost forms of predication whose syntactic framework are constituted by precisely these high-frequency, core functional dependencies. The concentration of network dependency types is, therefore, direct evidence of patients with Alzheimer’s disease being compelled to abandon diverse syntactic options in favor of a heavy reliance on basic existential constructions to state facts, thereby circumventing the increased cognitive load induced by long-distance dependencies. This over-reliance on the existential process comes at the expense of more complex transitive processes. The essence of transitivity, whether in a material process as in (5a), She’s [walking]…, or a mental process as in (5b), I [guess]…, requires the integration of multiple dependents around the verb. The syntactic realization of this multi-role integration is far more complex and cognitively demanding than a simple existential statement. The centralization of the AD dependency network is thus an adaptive reorganization of the grammatical system to avoid high-cost transitive structures.

(5) a. She’s [walking]_material process_ in the water. (AD Treebank)

b. I [guess]_mental process_ that’s for the under sink to put things. (AD Treebank)

Three key network metrics—Betweenness Centrality (C_B_), Closeness Centrality (C_C_), and Clustering Coefficient (C)—were subsequently introduced to quantify the structural function of nodes within the grammatical network (H. Liu, 2008b). T-tests on 30 core function-word nodes across the AD and HC networks revealed significant group differences for these parameters (see Table 8 and Table 9), indicating that the internal organizational architecture of the AD grammatical system has undergone a significant reorganization. Within the linguistic system of patients with AD, as the capacity to construct complex predicate structures around content words (especially verbs) atrophies, the entire structural load of syntactic connectivity is disproportionately transferred onto the remaining function words. Function words are thus transformed from efficient connectors in a healthy grammatical network into over-leveraged syntactic components in the AD network.

C_B_ measures the extent to which a node serves as a bridge between other nodes in the network. In the AD network, the disappearance of complex clausal structures compels connections that could once have been routed through multiple syntactic pathways to pass through a limited set of core function words (e.g., prepositions, conjunctions). Consequently, the betweenness centrality of these function words is necessarily elevated, as they become the most effective channels for linking otherwise isolated semantic blocks. The differences in C_C_ corroborate this finding: function words are forced to assume the role of central hubs in the AD network to maintain a minimal level of syntactic connectivity, preventing the network from devolving into disconnected lexical islands. Conversely, the centrality metrics for function words in the HC group were significantly lower. This does not imply that function words are unimportant in healthy language; on the contrary, it attests to the highly efficient and distributed nature of a healthy grammatical network. In the HC network, the syntactic load is effectively distributed across the entire grammatical apparatus, including complex verb phrases, clausal structures, and rich modifying elements. As a result, no single node type needs to function as the sole connection hub, and the network as a whole exhibits greater resilience and flexibility.

Furthermore, an analysis of local connectivity provides additional support for this thesis (see Table 10). The Clustering Coefficient reflects the degree to which a node’s neighbors are interconnected. The metrics for the AD group indicate that their function words tend to form highly localized, tightly knit syntactic structures. This aligns with the previous findings: to circumvent the demands of complex real-time planning, the AD grammatical system over-relies on retrieving and deploying prefabricated, fossilized phrasal modules. Function words become not only the framework connecting sentences but also the core and anchor of these high-frequency, low-cost structures.

The differences in network topological properties revealed in Table 10 systematically delineate how patients with Alzheimer’s disease reconfigure their syntactic strategies to maintain communicative function under conditions of constrained cognitive resources. The core of this strategy is an adaptive reliance on function words, behind which lies a syntactic reorganization pivoted on these elements. First, the structural importance of function words in the speech of patients with Alzheimer’s disease is significantly elevated. Their higher betweenness and closeness centralities jointly indicate that function words assume critical bridge roles within the dependency network. In the language of healthy controls, syntactic relations can be realized through complex hierarchical embedding (e.g., multiple clauses and modifiers); the AD network topology, however, shows that function words have become the default pathway for connecting syntactic constituents. They effectively lower the cognitive cost of constructing and parsing complex syntactic relations by shortening the dependency paths between words. This structural centralization reflects a shift from hierarchical embedding to linear concatenation. patients with Alzheimer’s disease tend to abandon cognitively costly syntactic operations, instead using function words like prepositions and conjunctions as grammatical markers to linearly string together simple chunks, thereby preserving information transmission within the limits of their cognitive resources.

Second, the local structural characteristics of the function word network reveal the specific morphology of this syntactic simplification. The higher clustering coefficient in the AD group signifies that function words and their immediate neighbors tend to form highly cohesive local network clusters. This finding, in conjunction with the slightly increased mean dependency distance, points to a specific compensatory mechanism. Function words no longer act merely as ordinary nodes but become the anchors for patterned, reusable lexical bundles. For instance, patients with Alzheimer’s disease frequently use fixed prepositional phrases or simple coordinate structures. These bundles, being internally cohesive, form stable linguistic units that are easily retrieved and deployed. When required to connect more distant sentence constituents (corresponding to an increase in dependency distance), patients deploy these formulaic bundles as bridges, thereby circumventing the cognitive burden of generating novel and complex long-distance dependencies. This explains why the speech of patients with Alzheimer’s disease can appear coherent at a macro level while exhibiting structural repetition and syntactic simplification at a micro level.

In contrast, the HC linguistic system does not primarily rely on a vast, pre-stored phrasal inventory. Instead, HCs utilize a finite set of lexical items and grammatical rules to generate language in real time. This computational process means that the neighboring nodes of a given function word are highly variable and unpredictable across different contexts. It might connect to a noun or to a variety of other complex phrases. This high degree of combinatorial possibility and flexibility directly inhibits the formation of stable, recurring clusters among neighboring nodes. Therefore, the sparse local connectivity pattern around function words in the HC network indicates that the HC system prioritizes expressive flexibility and novelty over retrieval efficiency. For the cognitively unimpaired brain, the cost of instant generation of complex structures is entirely manageable, making it a far more powerful and adaptive linguistic strategy than a reliance on fixed phrases.

To recapitulate, the HC network topology reflects a highly optimized system governed by the core principles of hierarchical generation and dynamic combination. Its distributed, low-clustering characteristics are a direct manifestation of this system’s dynamism and flexibility. In contrast, the systematic variations in the network topology of function words in the speech of patients with Alzheimer’s disease point collectively to a profound mechanism of linguistic adaptation. This is not a stochastic, global degradation of linguistic capacity, but rather a strategic reallocation of syntactic resources. Confronted with diminished working memory and cognitive resource capacity, the AD linguistic system achieves effective communication under the principle of cognitive economy by reinforcing the centrality of function words and shifting the locus of syntactic construction from the generation of complex hierarchical structures to the retrieval of patterned, linear ones. Therefore, the prominence of function words is a necessary, compensatory grammatical strategy adopted by patients with Alzheimer’s disease under pathological conditions to preserve linguistic coherence and structural integrity.

## 5. Conclusions

This study, through a deep analysis of dependency treebanks and networks, systematically reveals the syntactic restructuring mechanism in the language production of patients with Alzheimer’s disease. This restructuring is not a random degradation of linguistic ability but a profound, compensatory grammatical adaptation to preserve communicative function under the severe constraints of diminished working memory resources. The core findings can be summarized on four interconnected, progressively deeper levels. First is the strategic shift from hierarchical to linear complexity. The central change in the syntactic complexity of patients with AD is a fundamental transition from a preference for using clauses to construct complex, hierarchical information to a reliance on phrasal elaboration for linear organization. This shift is a direct projection of working memory deficits, driven by the need to avoid the high cognitive load of building and maintaining long-distance, cross-hierarchical dependencies. MDD serves as a precise quantitative index of this cognitive load, clearly delineating the intrinsic logic of the syntactic strategies employed. Second is the resource reallocation toward localized compensatory mechanisms. To achieve the aforementioned shift, the grammatical system of patients with Alzheimer’s disease undergoes a profound reallocation of resources. This is specifically manifested in the systematic avoidance of long-distance, cross-clausal dependencies marked by subordinating conjunctions, which significantly increase MDD. Concurrently, there is a prioritized and over-reliant use of cognitively less costly local dependencies as a compensatory measure. This includes adjectival modification within noun phrases, core predicate structures formed by verbs and direct objects, and the use of the existential there-construction as a syntactic framework. These localized strategies all point to a unitary principle: satisfying syntactic dependencies as locally as possible to minimize long-distance demands on working memory.

Third is the systemic reorganization from a distributed to a centralized network. The long-term application of these strategies ultimately leads to a systemic reorganization of the entire syntactic network topology. The grammatical network of healthy controls (HCs) presents as a highly efficient and flexible “distributed” system, where the syntactic load is shared among various components, including complex verb phrases and clauses. In contrast, the network of patients with Alzheimer’s disease degenerates into a “centralized” system, a key feature of which is the “over-leveraging” of function words. Due to the atrophy of complex predicate structures, function words are forced to assume the core roles of “bridges” in the network. Their betweenness and closeness centralities are significantly elevated, making them the essential channels for connecting isolated semantic blocks and maintaining a baseline of structural coherence. Finally, the outcome is an adaptive cost reflecting functional trade-offs under the principle of cognitive economy. The syntactic profile of patients with Alzheimer’s disease is the result of a functional balance driven by the principle of cognitive economy. Their linguistic system is not severely impaired; rather, it sacrifices expressive richness, flexibility, and grammatical depth in exchange for the possibility of maintaining basic information transmission under extremely limited cognitive resources. From syntactic simplification to network centralization, each step of this restructuring is a necessary, compensatory grammatical strategy adopted to preserve the structural integrity of language. Therefore, the syntactic signature of AD is not a collection of “errors” but profound linguistic evidence of a cognitive system adapting to survive under extreme cognitive constraints.

This study provides a more comprehensive linguistic description of the grammatical impairments in AD, yet the generalizability of its findings is subject to certain limitations. First, the age range of the participants in the corpus was broad and not highly concentrated, which may have introduced uncontrolled age-related variables that could affect the precision of the conclusions. Second, the demographic information for some participants, particularly years of education, was not fully documented, making it impossible to completely rule out the potential influence of educational background on syntactic complexity. Third, the reliance on the single “Cookie Theft” picture description task may limit the generalizability of our findings. Although this task was methodologically crucial for minimizing memory load and isolating syntactic changes, future research should examine how syntactic performance varies across a spectrum of discourse tasks with different cognitive demands (e.g., personal narratives). To further enhance reliability, future studies should also aim to construct more rigorously controlled and comprehensively annotated corpora. A related limitation is that the study’s scope was focused on validating the utility of these syntactic markers rather than establishing specific diagnostic thresholds for clinical screening. While defining such cut-off values is a critical step toward clinical application, it fell beyond the objectives of this foundational work and represents a key direction for future investigation. Finally, future research should prioritize the development of computational linguistic tools for automatic identification and quantification of the complex metrics employed in this study, alongside systematic evaluation of language intervention training effects on syntactic decline.

## Figures and Tables

**Table 1:** Dependent pairs and dependency distances of the sample sentence.

Dependent	Position	Governor Position	Part-of-Speech	Dependency Type	Dependency Distance
the	1	3	DT	det	2
young	2	3	JJ	amod	1
lad	3	5	NN	nsubj	2
is	4	5	VBZ	aux	1
going	5	0	VBG	root	0
to	6	7	TO	mark	1
fall	7	5	VB	xcomp	2
from	8	10	IN	case	2
the	9	10	DT	det	1
stool	10	7	NN	obl	3
.	11	5	PUNCT	punct	6

Note: Table 1 is the dependency deconstruction of the sample sentence.

**Table 2:** Indices referred to in the current study.

Name of Indices	Description
***Regarding*** ***Phrase Complexity***	
av_pobj_deps_NN	dependents per object of the preposition (no pronouns)
amod_all_nominal_deps_NN_struct	adjectival modifiers per nominal (no pronouns)
amod_dobj_deps_struct	adjectival modifiers per direct object
av_ncomp_deps_NN	dependents per nominal complement (no pronouns)
nominal_deps_stdev	standard deviation of dependents per nominal
pobj_NN_stdev	standard deviation of dependents per object of the preposition (no pronouns)
poss_nsubj_deps_NN_struct	possessives per nominal subject (no pronouns)
prep_all_nominal_deps_struct	prepositions per nominal
prep_nsubj_deps_NN_struct	prepositions per nominal subject (no pronouns)
rcmod_nsubj_deps_NN_struct	relative clause modifiers per nominal subject (no pronouns)
vmod_all_nominal_deps_NN_struct	verbal modifiers per nominal (no pronouns)
***Regarding*** ***Clause Complexity***	
cc_per_cl	clausal coordinating conjunctions per clause
ccomp_per_cl	clausal complements per clause
cl_av_deps	dependents per clause
cl_ndeps_std_dev	standard deviation of dependents per clause
conj_per_cl	conjunctions per clause
dobj_per_cl	direct objects per clause
mark_per_cl	subordinating conjunctions per clause
prep_per_cl	prepositions per clause
xcomp_per_cl	open clausal complements per clause

Note: Table 2 shows the indicators that appear in the TAASSC software in this article.

**Table 3:** Descriptive statistics for MDD variation.

Corpus	MDD	SD	F	T	*p*	Cohen’s d
AD	2.56	0.294	0.256	−3.216	0.001 *	0.294
HC	2.67	0.294				

Note: * Significant at the 0.05 level.

**Table 4:** Description of AD MDDs with grammatical indices.

Indices	B	SE	β	VIF	R^2^	Adjusted R^2^
cl_ndeps_std_dev	0.441	0.059	0.404	1.307	0.699	0.665
prep_all_nominal_deps_struct	1.505	0.322	0.262	1.402		
av_ncomp_deps_NN	0.064	0.013	0.252	1.225		
mark_per_cl	0.879	0.264	0.178	1.275		
ccomp_per_cl	0.715	0.177	0.211	1.204		
amod_dobj_deps_struct	0.739	0.138	0.315	1.531		
amod_all_nominal_deps_NN_struct	−0.790	0.251	−0.179	1.444		
xcomp_per_cl	0.433	0.180	0.120	1.093		
poss_nsubj_deps_NN_struct	−0.241	0.103	−0.116	1.089		
vmod_all_nominal_deps_NN_struct	−1.018	0.379	−0.138	1.166		
pobj_NN_stdev	0.144	0.046	0.164	1.213		
dobj_per_cl	−0.299	0.094	−0.165	1.214		
rcmod_nsubj_deps_NN_struct	0.479	0.149	0.158	1.073		
conj_per_cl	0.510	0.212	0.123	1.164		
prep_nsubj_deps_NN_struct	0.279	0.140	0.109	1.347		

Note: Table 4 represents the data description of the statistical results of patients with Alzheimer’s disease. B = Regression Coefficient, it represents the unstandardized slope of the regression line, indicating the change in the dependent variable for a one-unit change in the independent variable, holding other variables constant; SE = Standard Error, measures the variability or precision of the regression coefficient (B); β = Standardized Regression Coefficient; VIF = Variance Inflation Factor, measures the extent of multicollinearity among independent variables; R^2^ = R Square, indicates the proportion of variance in the dependent variable explained by the independent variables in the model, it ranges from 0 to 1, where a higher value suggests a better fit of the model to the data; Adjusted R^2^ measures the proportion of variance in the dependent variable explained by the independent variables, adjusted for the number of predictors in the model.

**Table 5:** Description of HC MDDs with grammatical indices.

**Indices**	**B**	**SE**	**β**	**VIF**	**R^2^**	**Adjusted R^2^**
cl_av_deps	0.401	0.071	0.408	2.193	0.662	0.643
av_pobj_deps_NN	0.136	0.048	0.164	1.382		
xcomp_per_cl	0.948	0.192	0.247	1.047		
cc_per_cl	−0.452	0.154	−0.172	1.443		
mark_per_cl	0.936	0.226	0.246	1.475		
conj_per_cl	0.842	0.266	0.177	1.299		
prep_per_cl	0.376	0.128	0.156	1.182		
nominal_deps_stdev	0.274	0.104	0.152	1.375		

Note: Table 5 presents the descriptive statistics for the HC group. The meaning of each index is the same as in Table 4.

**Table 6:** Top 50 dependency pairs of both AD and HC groups.

Cross Difference of N1 (1076)				Cross Difference of N2 (1136)			
Number of Vertices (n): 1076				Number of Vertices (n): 1136			
Arcs		Edges		Arcs		Edges	
Total Number of Lines 0 13,591				Total Number of Lines 0 15,979			
Density [Loops Allowed] = 0.02347777				Density [Loops Allowed] = 0.02476412			
Average Degree = 25.26208178				Average Degree = 28.13204225			
The Highest Values of Lines:				The Highest Values of Lines:			
1	1-6	prep	there-in	1	1-3	cc	it-and
2	1-6	prep	there-in	2	1-3	cc	it-and
3	1-6	prep	there-in	3	1-3	cc	it-and
4	1-6	prep	there-in	4	1-3	cc	it-and
5	1-6	prep	there-in	5	1-3	cc	it-and
6	1-6	prep	there-in	6	1-3	cc	it-and
7	1-6	prep	there-in	7	1-20	conj	it-sink
8	1-6	prep	there-in	8	1-21	nsubj	it-running
9	1-6	prep	there-in	9	1-21	nsubj	it-running
10	1-6	prep	there-in	10	1-21	nsubj	it-running
11	1-8	pobj	there-jar	11	1-21	nsubj	it-running
12	1-8	pobj	there-jar	12	1-22	nsubj	it-overflowing
13	1-12	nsubj	there-I	13	1-22	nsubj	it-overflowing
14	1-15	expl	there-’s	14	1-22	nsubj	it-overflowing
15	1-15	expl	there-’s	15	1-22	nsubj	it-overflowing
16	1-15	expl	there-’s	16	1-22	nsubj	it-overflowing
17	1-15	expl	there-’s	17	1-22	nsubj	it-overflowing
18	1-15	expl	there-’s	18	1-22	nsubj	it-overflowing
19	1-15	expl	there-’s	19	1-34	nsubj	it-have
20	1-15	expl	there-’s	20	1-36	conj	it-stool
21	1-15	expl	there-’s	21	1-37	dobj	it-moved
22	1-15	expl	there-’s	22	1-44	conj	it-boy
23	1-15	expl	there-’s	23	1-44	conj	it-boy
24	1-15	expl	there-’s	24	1-46	prep	it-to
25	1-15	expl	there-’s	25	1-46	prep	it-to
26	1-15	expl	there-’s	26	1-46	prep	it-to
27	1-15	expl	there-’s	27	1-54	nsubj	it-’s
28	1-15	expl	there-’s	28	1-54	nsubj	it-’s
29	1-15	expl	there-’s	29	1-54	nsubj	it-’s
30	1-15	expl	there-’s	30	1-54	nsubj	it-’s
31	1-15	expl	there-’s	31	1-54	nsubj	it-’s
32	1-15	expl	there-’s	32	1-54	nsubj	it-’s
33	1-15	expl	there-’s	33	1-54	nsubj	it-’s
34	1-15	expl	there-’s	34	1-54	nsubj	it-’s
35	1-15	expl	there-’s	35	1-54	nsubj	it-’s
36	1-15	expl	there-’s	36	1-54	nsubj	it-’s
37	1-15	expl	there-’s	37	1-54	nsubj	it-’s
38	1-15	expl	there-’s	38	1-54	nsubj	it-’s
39	1-15	expl	there-’s	39	1-54	nsubj	it-’s
40	1-15	expl	there-’s	40	1-54	nsubj	it-’s
41	1-15	expl	there-’s	41	1-54	nsubj	it-’s
42	1-15	expl	there-’s	42	1-54	nsubj	it-’s
43	1-15	expl	there-’s	43	1-54	nsubj	it-’s
44	1-15	expl	there-’s	44	1-54	nsubj	it-’s
45	1-15	expl	there-’s	45	1-54	nsubj	it-’s
46	1-15	expl	there-’s	46	1-54	nsubj	it-’s
47	1-15	expl	there-’s	47	1-54	nsubj	it-’s
48	1-15	expl	there-’s	48	1-54	nsubj	it-’s
49	1-15	expl	there-’s	49	1-54	nsubj	it-’s
50	1-15	expl	there-’s	50	1-54	nsubj	it-’s

Note: Table 6 shows the top fifty dependent pairs of AD Group and the HC group after the Cross Difference step in Pajek.

**Table 7:** Top 30 weighted vertices in both AD and HC networks.

Weighted All Degrees of N3 (107)				Weighted All Degrees of N4 (127)			
Dimension: 107				Dimension: 127			
The Lowest Value: 0.0000				The Lowest Value: 1.0000			
The Highest Value: 31.0000				The Highest Value: 41.0000			
Density: 0.03493755				Density: 0.02467605			
Average Degree: 3.73831776				Average Degree: 3.13385827			
Rank	Vertex	Value	Id	Rank	Vertex	Value	Id
1	1	31	there	1	1	41	it
2	19	15	is	2	4	17	the
3	6	13	I	3	8	13	a
4	17	13	the	4	3	11	and
5	4	12	and	5	7	11	there
6	66	11	jar	6	19	9	to
7	12	10	it	7	2	9	was
8	67	10	girl	8	67	8	sink
9	46	9	be	9	93	7	house
10	2	9	a	10	78	7	jar
11	64	9	’s	11	75	7	stool
12	100	8	see	12	68	7	overflowing
13	18	8	that	13	6	6	in
14	3	7	in	14	30	5	or
15	25	7	this	15	26	5	is
16	96	7	apron	16	97	5	’s
17	76	7	know	17	95	5	seeing
18	68	7	saying	18	87	5	going
19	97	6	window	19	83	5	place
20	48	6	something	20	60	4	window
21	20	6	on	21	29	4	that
22	75	6	sink	22	59	4	open
23	73	6	overflowing	23	116	4	fact
24	15	5	to	24	115	4	oblivious
25	28	5	more	25	105	4	see
26	94	5	told	26	104	4	garden
27	92	5	kitchen	27	11	4	did
28	83	5	think	28	46	4	are
29	79	5	wife	29	10	4	they
30	34	5	she	30	21	4	his

Note: Table 7 shows the top 30 weighted vertices in the discourse of AD Group and the HC group.

**Table 8:** Three parameters of functional words in AD and HC networks.

	AD		HC			
Vertex	C_B_	C_C_	C	C_B_	C_C_	C
there	0.022099	0.457022	0.121477	0.013476	0.414263	0.127551
you	0.009195	0.37477	0.060256	0.006968	0.343243	0.012315
so	0.003254	0.393383	0.141129	0.004128	0.372544	0.060504
this	0.03203	0.452823	0.080184	0.001157	0.394093	0.156085
here	0.022918	0.456552	0.120702	0.002895	0.391002	0.231579
whether	0.000529	0.331495	0.066667	0.000059	0.308672	0
from	0.003303	0.396018	0.157143	0.000227	0.341141	0.004762
down	0.006221	0.409358	0.123113	0.000378	0.341264	0.011696
may	0.003326	0.305573	1	0.000024	0.328479	0.166667
must	0.000249	0.338064	0.145455	0.000011	0.302655	0
was	0.008557	0.410305	0.107692	0.001198	0.383714	0.115942
should	0.000078	0.326492	0	0.000029	0.296495	0
where	0.001342	0.400126	0.205882	0.004412	0.378651	0.084967
some	0.011713	0.412212	0.1	0.004527	0.349707	0.054348
did	0.001305	0.408981	0.263158	0.00131	0.321128	0
onto	0.000019	0.344765	0.5	0.000013	0.302075	0
does	0.000006	0.362814	0.4	0.000007	0.31389	0
then	0.000542	0.397081	0.171429	0.000177	0.340527	0.051282
sometimes	0	0.316588	0	0	0.247907	0
very	0.008912	0.322574	0.012821	0.002228	0.282688	0
mostly	0	0.320941	0	0.000087	0.27011	0
after	0.002219	0.375563	0.333333	0.000001	0.311515	0
my	0.002512	0.336015	0.054545	0.000029	0.277714	0
those	0.000325	0.353418	0.285714	0.000006	0.291028	0
someone	0.000006	0.331495	0.2	0	0.262111	0
could	0.000247	0.371163	0.181818	0.000247	0.329738	0
pretty	0.003071	0.351319	0.133333	0.002493	0.315039	0
through	0.000473	0.354974	0.075758	0.000001	0.308572	0
neither	0	0.299993	0	0	0.22225	0
unless	0	0.290465	0	0	0.210821	0

Note: Table 8 shows the betweenness centrality, closeness centrality and clustering coefficient of the vertices of functional words in the dependency networks of AD Group and the HC group. C_B_ = betweenness centrality; C_C_ = closeness centrality; C = clustering coefficient.

**Table 9:** Descriptive statistical differences between the AD and HC groups.

	F	t	*p*	Cohen’s d
C_B_	11.136	2.133	0.037	0.0060
C_C_	0.008	3.906	0.000	0.04790
C	6.880	3.477	0.001	0.1472

Note: Table 9 shows the descriptive statistical differences in betweenness centrality, closeness centrality and clustering coefficient of the dependency network between AD Group and the HC group. F = F-statistic, is used in regression analysis to test the overall significance of the model; t = t-statistic, is used in regression analysis to assess the significance of individual predictors or group differences; Cohen’s d is a measure of effect size, quantifying the standardized difference between two means, typically used to assess the magnitude of differences.

**Table 10:** Group statistics of C_B_, Cc and C in AD and HC groups.

	Group	N	Mean	SD	SE Mean
C_B_	AD	30	0.0048	0.0079	0.00144
	HC	30	0.0015	0.0029	0.00053
Cc	AD	30	0.3667	0.0464	0.00847
	HC	30	0.3184	0.0493	0.00901
C	AD	30	0.1681	0.1985	0.03624
	HC	30	0.0359	0.0626	0.01142

Note: Table 10 shows the descriptive statistical differences in betweenness centrality, closeness centrality and clustering coefficient of the entire dependency network between AD group and the HC group.

## Data Availability

The corpus data used in this study is available through the DementiaBank database, contingent upon successful registration and authorization. It is essential that the data source be properly cited in any resulting publications. The initial acquisition of the DementiaBank database was supported by NIH grants AG005133 and AG003705, awarded to the University of Pittsburgh. Additional details regarding access and usage can be found at https://dementia.talkbank.org/ (accessed on 9 January 2025).

## Abbreviations

The following abbreviations are used in this manuscript:

AD	Alzheimer’s disease
MDD	mean dependency distance
DD	dependency distance
HC	healthy control
MSL	mean sentence length
TTR	type-token ratio
